# Editorial: Wearable Sensor Technology for Monitoring Training Load and Health in the Athletic Population

**DOI:** 10.3389/fphys.2019.01520

**Published:** 2020-01-08

**Authors:** Billy Sperlich, Kamiar Aminian, Peter Düking, Hans-Christer Holmberg

**Affiliations:** ^1^Integrative and Experimental Exercise Science & Training, University of Würzburg, Würzburg, Germany; ^2^Laboratory of Movement Analysis and Measurement, Ecole Polytechnique Fédérale de Lausanne, Lausanne, Switzerland; ^3^Department of Health Sciences, Mid Sweden University, Östersund, Sweden; ^4^School of Kinesiology, University of British Columbia, Vancouver, BC, Canada; ^5^Department of Physiology and Pharmacology, Biomedicum C5, Karolinska Institutet, Stockholm, Sweden

**Keywords:** wearables, data analysis, personalized medicine, monitoring, sensor, biofeedback, innovation, digital health

Various measures of the internal and external loads on athletes, as well as parameters related to their health are now being provided to a greater and greater extent by wearable sensors (wearables) (Düking et al., 2018a,b,c). These devices, including sensors and software embedded in e.g., textiles, watches and patches located on or in proximity to the body, collect, transmit, and analyse a range of physiological and biomechanical data designed to improve performance, recovery, and/or other aspects of health (Düking et al., 2018a). However, it is still unclear to what extent wearables are actually useful for monitoring load in connection with different sports and settings.

In 2017, we launched a special coverage of the Research Topic “Wearable Sensor Technology for Monitoring Training Load and Health in the Athletic Population” in *Frontiers in Physiology* with the following aims:

- to identify and critically evaluate promising wearable technology designed to monitor training load and health in athletic populations;
- to develop novel approaches to data analysis based on advanced modeling, time series, machine learning, data mining, etc.;
- to encourage the use of (best-practice) models for monitoring training load and health in athletes; and
- to indicate directions for future development in this area.

One hundred thirteen authors have now published 28 articles in *Frontiers in Physiology* on this Research Topic, including 18 original articles based on field and laboratory data, four (mini) reviews, three opinion papers, one perspective and one technology report. Table 1 summarizes the main features of all of these studies. With more than 148,000 views (as of November 2019), this Research Topic is among those published in the Physiology section of *Frontiers in Physiology* that have received most interest. To achieve the aims described above, we have grouped these articles in the table on the basis of the specific sport involved or evaluation of new technologies without consideration of any specific population, describing only those articles we consider to be of primary importance in the field.

**Table 1:** Summary of all studies within the Research Topic including type of article, athletes involved, sensors employed and main outcome.

	**References**	**Type of article**	**Athletes involved**	**Sensor(s) employed**	**Research aim(s)**	**Main outcome(s)**
Evaluation of the Quality of New Sensor Technology	Peake et al.	Review	Physically active individuals	Consumer-grade wearables	A critical review of consumer wearables, mobile applications, and equipment for providing biofeedback, monitoring stress, and sleep.	So far, only 5% of the technologies have been validated formally. Companies producing health and performance technologies should consult with consumers to identify real-world needs and invest in research designed to confirm the effectiveness of their products.
	Koehler and Drenowatz	Mini-review	n.i.	SenseWear armband	Providing an overview of the applicability of this sensor.	Estimates energy expenditure by individuals in the general population reliably. Application to athletic populations indicates a tendency to underestimate energy expenditure.
	Wahl et al.	Original research	Healthy students	11 Wrist-worn wearables (Bodymedia Sensewear, Beurer AS 80, Polar Loop, Garmin Vivofit, Garmin Vivosmart, Garmin Vivoactive, Garmin Forerunner 920XT, Fitbit Charge, Fitbit Charge HR, Xiaomi Mi Band, Withings Pulse Ox)	Validation of the reliability of 11 wearables for monitoring step count, distance covered and energy expenditure (EE) under laboratory conditions at constant and different velocities.	The accuracy of most of these wearables is acceptable for counting steps at constant and intermittent running velocities that reflect athletic activities. With respect to distance covered, all of these wearables exhibited a very low ICC (<0.1) and high MAPE (up to 50%), indicating poor validity. Measurement of EE by the Garmin, Fitbit and Withings wearables was acceptable (small to moderate MAPE), whereas Bodymedia Sensewear, Polar Loop, and Beurer AS80 showed a high MAPE (as much as 56%) under all test conditions.
	Sorbie et al.	Original research	n.i.	Myon 320 Surface Electromyography (sEMG) System	To determine the intra-session and inter-day reliability of this system.	Intra- and inter-day measurement of the normalized root-mean-squared surface EMG is reliable during dynamic sub-MVC, when exercise is performed at low velocities.
Wearables in Connection with Team Sports	Mascarin et al.	Original research	Soccer players	n.i.	To analyse hormonal, biochemical, and autonomic parameters during a game with few players, as well as recovery dynamics (for up to 72 h).	Such games involved significant cardiovascular stress that returned to baseline within the first 24 h of recovery. Parasympathetic parameters continued to increase while sympathetic parameters declined significantly during the 72 h of recovery. Neither biochemical nor hormonal responses were altered during these 72 h.
	Pettersen et al.	Brief research report	Soccer players	A wearable, radio-based system for determining position	To highlight some of the challenges encountered when using positional data in research, as well as for team development, and to propose other promising sources of data.	Measurement of the locomotion of a player with existing positional technologies is not always accurate.
	Fuss et al.	Original research	Soccer players	Smart Soccer Boot Sensor	Application of this technology for exploring the accuracy of curved kicks. Evaluation of the relationship between the probability of scoring a goal, and dynamic parameters provided by the smart boot. Determination of whether kicking the ball at a particular spot on the shoe (“sweet spot”) maximizes the chances of success.	With a stationary curved kick, kicking the ball with the “sweet spot” of the shoe maximized the probability of scoring a goal (58–86%).
	Roell et al.	Original research	Members of Indoor sports teams	Inertial Measurement Unit	To validate this technology regarding determination of average and peak acceleration during sport-specific movements.	MEMS-based sensors exhibit great potential for valid determination of acceleration and deceleration during specific movements associated with indoor sports, including walking, running, jumping, and change of direction.
	Weaving et al.	Opinion	Members of sports teams	n.i.	n.i.	This multivariate approach warrants further investigation, at least initially for research purposes, in light of the importance of assessing training load in attempt to optimize preparation by team-sport players.
	Schneider et al.	Technology report	Members of sports teams	Heart rate monitors	Describe current limitations of heart rate monitoring. Discuss methodological considerations associated with univariate and multivariate approaches. Illustrate the influence of different analytical concepts on the assessment of meaningful alterations in heart rate. Provide examples of the contextualization of heart rate measures based on simple heuristics.	The conceptual framework developed contextualizes measures of heart rate, focusing on the time-course of training responses, as well as the training context. In addition, heuristic application of this framework to multivariate interpretation and decision-making is illustrated.
	Luteberget et al.	Original research	Members of sports teams	Local positioning systems (LPS)	Validate the measurement of the position, distance traveled and instantaneous speed of players during indoor team sports by a commercially available LPS Investigate how the position of the field of play relative to the anchor nodes and walls of the building influence the validity of this system.	The mean difference between the estimate of all positions by the LPS and reference system was 0.21 ± 0.13 m (*n* = 30,166) with the optimal setup and 1.79 ± 7.61 m (*n* = 22,799) with the sub-optimal setup. The average difference in distance during all tasks was <2% with the optimal setup and <30% with the sub-optimal setup. Among all of the variables assessed, the difference in instantaneous speed as indicated by the LPS and reference system was largest, both in the optimal (≥35%) and sub-optimal condition (≥74%). Moreover, this difference increased with increasing speed.
	Sweeting et al.	Review article	Members of sports teams	GPS LPS Based on vision	Identify the various thresholds used to classify high-velocity or -intensity running and acceleration. Examine the impact of individualized thresholds on the reported profile of team-sport activity. Evaluate the usefulness of	It is difficult to compare research findings on field-based sports due to the use of different velocity and acceleration thresholds, even in the case of one and the same sport. Research on female team-sport athletes, including how to classify their velocity and acceleration, is limited.
					thresholds for court-based team sports. Discuss potential areas for fruitful future research.	Data mining can provide further insights concerning athletic activities.
	Nikolaidis et al.	Original research	Track-and-field athletes and female soccer players	10-Hz Global Positioning System	Examine the validity and reliability of this system for assessing in-line movement and change of direction.	The 10-Hz Johan GPS system provides valid and reliable monitoring of team-sport players and endurance runners during training with respect to linear movement and change-of-direction.
	Corbett et al.	Original research	Australian soccer players	n.i.	Identify how an athlete's skills change as a football match proceeds. Determine the extent to which these changes are due to individual factors. Reveal the relationship between various combinations of physical and skill parameters and performance.	Two methods for identifying relationships between physical, skill and temporal parameters of both the individual players and team as a whole were developed.
Wearables in Connection with Winter Sports	Fasel et al.	Original research	Alpine ski racers	Inertial sensors	Gain a more in-depth understanding of the relationship between external training load and health.	The system determines the athlete's relative joint center and center of mass (CoM) with sufficient accuracy and precision to allow detection of meaningful differences. Accuracy and precision regarding the most distal joints (e.g., the ankle) are just within the acceptable range. For example, the ankle positions obtained allow acceptable computation of the vertical distance, but not of the fore-aft position.
	Spörri et al.	Original research	Alpine ski racers	Inertial Measurement Units	Describe the power spectral density (i.e., the power of the signal in relationship to frequency) of the vibrations acting on different body segments during giant slalom and slalom competitions. Quantify and compare root-mean-square acceleration of the lower back during the turns involved in these disciplines.	In addition to combined frontal and lateral bending, along with torsion in the trunk when highly loaded, vibrations acting on the lower back may contribute to overuse injuries of the back.
	Supej et al.	Original research	Alpine skiers	Accelerometer Global Navigation Satellite System (GNSS)	Examine the whole-body vibrations associated with different types of skiing and the potential risk these pose for developing lower-back pain.	All forms of skiing tested produced whole-body vibrations, with highest power spectrum densities of 1.5–8 Hz. Whole-body vibrations, particularly in combination with high ground reaction forces, elevate the risk for lower- back pain among active alpine skiers.
	Gilgien et al.	Original research	Alpine ski racers	Differential GNSS	Characterize the external forces acting on competitors in World Cup Super-G and downhill ski races and compare these to those connected with giant slalom racing.	These systems are sufficiently reliable to allow meaningful characterization of physical demands, as well as the effectiveness of safety measures in connection with highly dynamic sports. The physical demands associated with giant slalom,
						super-G and downhill ski races differed significantly. Reduction of skiing speed during giant slalom and super-G races for purposes of safety might be achieved most effectively by increasing ski–snow friction.
	Karlsson et al.	Original research	Cross-country skiers	GNSS	Investigate patterns of pacing by characterizing exercise intensity on flat and uphill terrain during a simulated cross-country ski race.	Cross-country skiers perform repeatedly at exercise intensities that exceed their maximal aerobic power. O_2_ deficits were higher when skiing uphill than on flat terrain.
Wearables in Connection with Running and Cycling	Belbasis and Fuss	Original research	Cyclists	Smart compression garment containing a pressure sensor	Explore the potential of a prototype garment containing pressure sensors for analysis of performance, specifically of muscle activation and fatigue. Compare the data provided by this prototype to electromyography (EMG)	Assessment of muscle activity and fatigue by the smart compression garment was similar to that provided by surface EMG.
	Wouda et al.	Original research	Runners	Inertial sensors	Examine the validity of estimating sagittal knee joint angles and vertical ground reaction forces while running a minimal setup involving sensors worn on the body.	Sagittal knee kinematics and vertical ground reaction forces can be estimated reliably using as few as 3 inertial sensors located on the lower legs and pelvis. In particular, the peak vertical ground reaction force, maximal knee flexion/extension angles while standing, and profiles of knee flexion/extension angles and vertical ground reaction forces estimated in this manner do not differ significantly from reference values.
	Falbriard et al.	Original research	Healthy adults	Inertial sensors worn on the foot	Assess the ability of various kinematic measures provided by foot-worn inertial sensors to detect temporal events during running (e.g., initial and terminal contact) and thereby allow estimation of the duration of different phases (e.g., contact, flight, swing, step).	Ground contact, flight, step and swing times can all be estimated with a median inter-trial IQR bias of <12 ± 10 ms and an error <4 ± 3 ms. Running speed significantly affects the bias of these estimations, indicating that a speed-dependent correction can improve accuracy.
Other	Reed et al.	Original research	Nurses working with cardio-vascular patients	Tractivity^®^ activity monitor	Examine the impact of providing feedback from an activity monitor, in combination with a web-based individual, friend or team challenge, on physical activity and cardiovascular health.	This intervention had an initial impact on physical activity, percentage body fat and resting systolic BP, although the increase in physical activity was short-lived. The nature of the challenge had no influence on the outcome.
	Düking et al.	Opinion	n.i.	n.i.	n.i.	Biofeedback may help optimize training and health.
	Nicolò et al.	Perspective	n.i.	n.i.	Provide scientific evidence for the relevance of monitoring respiratory frequency in connection with training. Critically assess potential approaches to measuring respiratory frequency, as well as the accuracy of respiratory wearables currently available. Provide preliminary indications concerning how best to analyse respiratory frequency.	Scientific evidence supports the usefulness of monitoring respiratory frequency during training. Potential approaches involving wearable sensors currently available are proposed. Indications concerning how best to analyse and interpret respiratory frequency data are provided.
	Ludwig et al.	Review	n.i.	Heart rate sensors	Provide an overview of the measurement, prediction, and control of individual changes in heart rate. Analyse heart rate sensors presently available regarding their responses and feasibility.	Wearables that monitor individual load on the basis of heart rate are of potential value in connection with physical training.
	Gløersen et al.	Original research	Endurance racers	GNSS	Assess the accuracy of three different classes of GNSS receivers for measuring position, speed, and segment time during endurance races.	There are substantial differences in the accuracy of different GNSS receivers. The split-time error was strongly dependent on (and inversely related to) the athlete's speed. The segment-time error was greater for longer segments.

*n.i., not indicated*.

## Evaluation of the Quality of New Technology

Peake et al. critically reviewed consumer-grade wearables, mobile applications, and equipment designed to provide biofeedback to physically active individuals. While acknowledging that wearable technology has much to offer, these investigators concluded that only 5% of the technologies they reviewed have been formally validated and that manufacturers should invest in studies on the effectiveness of their products.

Wahl et al. showed that under different sporting conditions, the majority of 11 wrist-worn wearables demonstrated acceptable validity with respect to counting steps, whereas the distance covered and energy expenditure could not be assessed validly.

Reviewing the relevant literature, Koehler and Drenowatz concluded that while the SenseWear armband can estimate energy expenditure validly in the general population, it tends to underestimate this parameter during high-intensity exercise (>10 METs).

## Wearables in Connection with Winter Sports

Wearables are often utilized to assess parameters associated with different skiing disciplines. Employing a global navigation satellite system, Karlsson et al. found that cross-country skiers repeatedly perform at intensities that exceed their maximal aerobic power, with more pronounced oxygen deficits during uphill skiing than on flat terrain.

Gilgien et al. applied a differential global navigation satellite system (dGNSS) to evaluate the physical demands and safety associated with different skiing disciplines. The physical demands made by giant slalom, super-G and downhill skiing differ substantially. Furthermore, these researchers concluded that to increase safety, skiing speed can best be reduced by enhancing the friction between the skis and snow and in the case of giant slalom and super-G, whereas for downhill skiing an elevation in air drag force might be equally effective.

Using five accelerometers and a global navigation satellite system, Supej et al. found that low-frequency whole-body vibrations during alpine skiing enhance the risk for pain in the lower back, particularly in combination with large ground reaction forces. They concluded that the number of runs involving such vibrations (e.g., during side-skidding) should be reduced, especially in the case of younger skiers.

Spörri et al. evaluated vibrations acting on different body segments during giant slalom and slalom skiing with 6 wearable inertial measurement units. Power distribution over frequency (PSD) was largest with frequencies of <30 Hz in the case of the shank, with vibrations being attenuated by the knee and hip joints. PSD values were pronounced at frequencies between 4 and 10 Hz, increasing the risk of overuse back injuries in alpine skiers.

Applying 11 inertial measurement units, Fasel et al. could assess the kinematics of the relative center of mass and positions of joint centers of alpine skiers with sufficient accuracy and precision, while the ankle joints were only just within the acceptable range of accuracy and precision.

## Wearables in Connection with Team Sports

In their original article, Fuss et al. employed a pressure-sensitive sensor matrix incorporated into a soccer shoe to identify a “sweet spot” on the foot that maximizes the chances of hitting the goal with a direct curved free kick of 58–86°. This sensor may allow soccer players to analyse their foot-to-ball impact and improve their technique.

In connection with team sports, tracking technologies, such as global positioning (GPS), local positioning (LPS), and vision-based (VBS) systems, allow activity profiles to be monitored. Analysis of these profiles may be influenced by the relative amount of time spent in different velocity or acceleration zones and Sweeting et al. emphasize in their review article that there is presently no generally accepted definition of a sprint or acceleration, not even within a given team sport, which complicates comparison of different studies.

With respect to training load, Weaving et al. argue that no single parameter is likely to capture the complexity of this parameter and, moreover, practitioners can be overwhelmed by the amount of data they receive. A multivariate approach employing selected orthogonal composite variables may be helpful in providing sufficient data without “flooding.”

For quantifying aspects of external loading in connection with indoor team sports, Roell et al. found a wearable inertial unit designed to measure average and peak acceleration to be acceptably valid in all three orthogonal axes.

In their case study, Pettersen et al. demonstrate that wearable radio-based positioning systems can provide insights into the performance of individual soccer players and their teams.

## Wearables in Connection with Running and Cycling

Belbasis and Fuss found that a pressure-sensitive sensor located inside compression garments provided data on the activity on five thigh muscles during cycling comparable to that obtained by electromyography (EMG). Arguably, this smart compression garment monitors mechanical muscle activity (i.e., the pressure exerted by the contracting muscle on the sensor), whereas EMG measures neural activity and may therefore be more suitable for biomechanical modeling.

In the case of runners, Falbriard et al. showed that temporal parameters, involving ground contact, flight, step, and swing times can be estimated accurately, but that the results obtained are dependent on the speed.

Wouda et al. found that estimation of the peak vertical ground reaction force, as well as maximal knee flexion-extension angles during stance in runners by three inertial measurement systems in combination with artificial neural networks did not differ significantly from the reference values.

## Concluding Remarks

The 28 articles on this Research Topic have clearly improved our knowledge concerning the use of wearables for monitoring training load and health in athletes involved in a different sports. Novel technologies have been introduced and technologies already existing evaluated. New approaches to monitoring and analyzing (training) load in connection with different sports have been described. Nonetheless, much remains to be determined concerning the usage of wearables by athletic populations.

While some findings involve physiological parameters, e.g., those of Nicolò et al., most of the wearable technology investigated provides biomechanical data. Therefore, we encourage future studies on physiological parameters in this area of research. Since future monitoring frameworks (Düking et al., 2018a) may provide instant feedback concerning internal load to coaches and athletes, such research is certainly warranted. Appropriate combination of physiological and psychological data with biomechanical data will be a future challenge in connection with providing relevant and seamless feedback to the athlete.

Currently, on the basis of the articles included here, it remains unclear whether monitoring with wearables is actually beneficial for controlling the load and improving the health of athletes. To date, no publication has addressed these questions directly.

Future advancements in smart technology will involve devices designed to share and interact with their users, as well as with other smart devices. Wearables should, however, be convenient and usable without hindering the athlete with cumbersome sensors. Optimal **i**ntegration of sensors into equipment (e.g., ski boots, garments) will require the involvement of manufacturers of sporting equipment.

Today, 3 years after we launched “Wearable Sensor Technology for Monitoring Training Load and Health in Athletes” as a Research Topic, interest remains quite high, as indicated, among other things, by global fitness trends (Thompson, 2019). We look forward to the novel insights arising from future research in this growing field.

## Author Contributions

BS, PD, KA, and H-CH wrote and edited the manuscript.

### Conflict of Interest

The authors declare that the research was conducted in the absence of any commercial or financial relationships that could be construed as a potential conflict of interest.
